# Ablation Behavior of the SiC-Coated Three-Dimensional Highly Thermal Conductive Mesophase-Pitch-Based Carbon-Fiber-Reinforced Carbon Matrix Composite under Plasma Flame

**DOI:** 10.3390/ma12172723

**Published:** 2019-08-25

**Authors:** Chong Ye, Dong Huang, Baoliu Li, Pingjun Yang, Jinshui Liu, Huang Wu, Jianxiao Yang, Xuanke Li

**Affiliations:** 1 College of Materials Science and Engineering, Hunan Province Key Laboratory for Advanced Carbon Materials and Applied Technology, Hunan University, Changsha 410082, China; 2 Hunan Province Engineering Research Center for High Performance Pitch Based Carbon Fiber, Hunan Toyi Carbon Material Technology Co., Ltd., Changsha 410000, China; 3 The State Key Laboratory of Refractories and Metallurgy, Wuhan University of Science and Technology, Wuhan 430081, China

**Keywords:** carbon–carbon composites, ablation, high-temperature properties, microstructure

## Abstract

This study is focused on a novel high-thermal-conductive C/C composite used in heat-redistribution thermal protection systems. The 3D mesophase pitch-based carbon fiber (CF_MP_) preform was prepared using CF_MP_ in the X (Y) direction and polyacrylonitrile carbon fiber (CF_PAN_) in the Z direction. After the preform was densified by chemical vapor infiltration (CVI) and polymer infiltration and pyrolysis (PIP), the 3D high-thermal-conductive C/C (C_MP_/C) composite was obtained. The prepared C_MP_/C composite has higher thermal conduction in the X and Y directions. After an ablation test, the CF_PAN_ becomes needle-shaped, while the CF_MP_ shows a wedge shape. The fiber/matrix and matrix/matrix interfaces are preferentially oxidized and damaged during ablation. After being coated by SiC coating, the thermal conductivity plays a significant role in decreasing the hot-side temperature and protecting the SiC coating from erosion by flame. The SiC-coated C_MP_/C composite has better ablation resistance than the SiC-coated C_PAN_/C composite. The mass ablation rate of the sample is 0.19 mg·(cm^−2^·s^−1^), and the linear ablation rate is 0.52 μm·s^−1^.

## 1. Introduction

Carbon-fiber-reinforced carbon matrix (C/C) composite has been widely studied due to its high-temperature strength, low coefficient of thermal expansion, and good anti-ablation ability [1,2]. Because of its excellent performance, C/C composite has become a promising structural material in high-temperature applications, including rocket nozzles, aeronautic jet engines, leading edges, and so on. C/C composite is also a promising functional material for thermal management systems due to its high thermal conductivity [3,4]. Because it can decrease the temperature of hot components and consequently increase their reliability, C/C composite has been used in heat-redistribution thermal-protection systems of hypersonic aircraft that have undergone long-time ablation and oxidation [5]. 

There have been many studies on the effects of voids, carbon fibers, and matrix and the interfaces between them on the mechanical and thermal performance of C/C composites. Lachaud et al. [6] set up a modeling strategy to predict 3D C/C composite ablation behavior, and the models were consistent with the experimental data. Lee et al. [7] characterized the surface microstructure of a 2D C_PAN_/C composite and found that the transverse CF_PAN_ of flank surface had a random orientation of basal planes that was different from the fiber surface. Baxter et al. [8] studied the effect of chemical vapor infiltration (CVI) on the corrosion and thermal conduction of porous C/C composite, and found that both radiative heat transfer and the heat transfer path in pores played important roles in the parallel path thermal conduction. The ablation behavior of C/C composite was also studied by other researchers [9,10]. As for high-thermal-conductive C/C composite, carbon fiber (CF) is not only the reinforcement of the composite, but also the carrier of heat [11]. The morphology, crystal orientation, and crystallite structure of the carbon fiber have an obvious influence on the heat transfer of C/C composite [12,13]. Polyacrylonitrile (PAN)-based and pitch-based carbon fibers are the most common carbon fibers [14,15]. In comparison with PAN-based carbon fiber (CF_PAN_), mesophase pitch-based carbon fiber (CF_MP_) has higher thermal conductivity due to fewer lattice defects, higher preferred orientation, and larger graphite crystallite size [15,16]. Therefore, CF_MP_ is an ideal reinforcement for C_MP_/C composite with high thermal conductivity. Many studies have focused on developing high-thermal-conductive C_MP_/C composites. Yuan et al. [17] prepared a 1D ribbon-shaped carbon-fiber-reinforced C_MP_/C composite, and the longitudinal thermal conductivity was about 900 W·(m^−1^ K^−1^) after graphitization. Adams et al. [18] and Manocha et al. [4] analyzed the microstructure and thermophysical properties of C_MP_/C composite. Although much data has been reported in the literature on the fabrication of high-thermal-conductive C_MP_/C composite [18] and there has been evaluation of the thermophysical properties [19,20] and analysis of the thermal conductivity mechanism [21,22], there have been few reports on the ablation behavior of C_MP_/C composite, which is worth exploring. It is known that the microstructures and properties of CF_MP_ and CF_PAN_ are evidently different. High-thermal-conductive CF_MP_ has been proven to have a perfect graphite crystal structure highly oriented along the fiber axis direction, while CF_PAN_ exhibits a turbostratic structure composed of entangled and wrinkled crystallites [13,16]. Therefore, it can be speculated that C_MP_/C composite using CF_MP_ as reinforcement will have some different ablation features. Such understanding of the ablation behavior of C_MP_/C composite is helpful in applying this composite to thermal protection systems of hypersonic vehicles, especially in explaining the failure mechanisms after the thermal protection coating is damaged. In addition, poor oxidation resistance of carbon materials (above 500 °C) has greatly restricted the application of C_MP_/C composite under high-temperature environments. Therefore, a thermal protective coating is also needed to make the high thermal conductivity play its positive role in ablation [23,24]. All of this should be experimentally investigated.

In this paper, we report the ablation behavior of a novel homemade 3D C_MP_/C composite with high thermal conductivity. The C_MP_/C composite was prepared using CF_MP_ as the reinforcement and heat carrier in the X (Y) direction and CF_PAN_ in the Z direction. The influence of thermal diffusion and structure on the ablation behavior of C_MP_/C composite with and without SiC coating is elucidated. This study can provide guidance for the design and fabrication of C_MP_/C composite with high thermal conductivity for application in hypersonic aircrafts.

## 2. Experimental Procedures

### 2.1. Material Preparation

**Figure 1:**
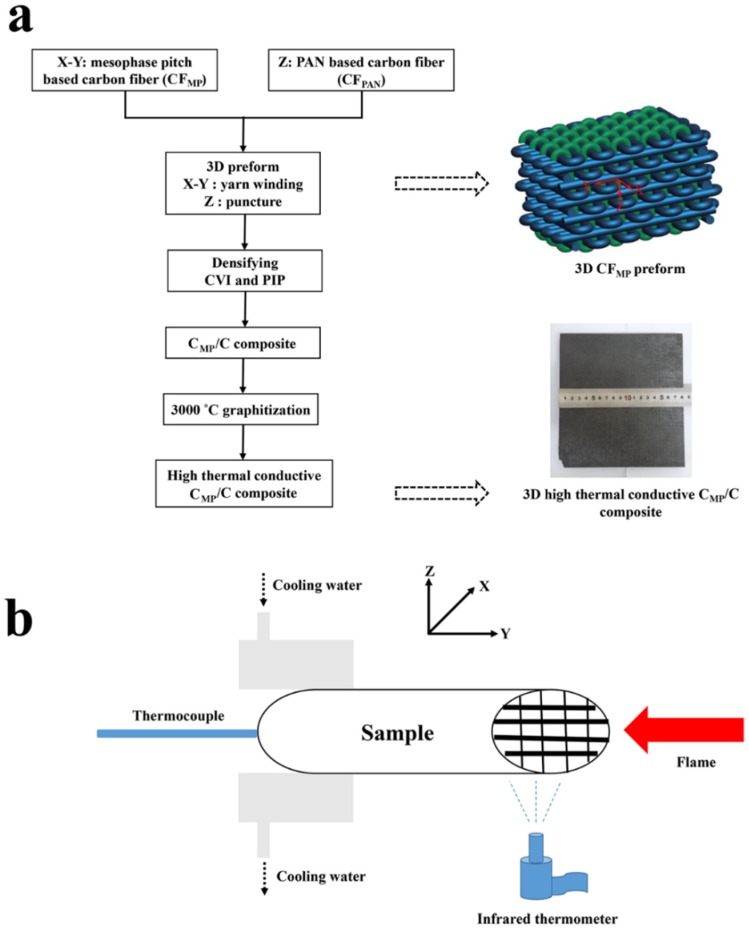
(**a**) Preparation process and (**b**) ablation test of C_MP_/C composite. CVI, chemical vapor infiltration; PIP, polymer infiltration and pyrolysis; C_MP,_ mesophase pitch-based carbon fiber.

The high-thermal-conductive C_MP_/C composite was prepared as follows (Figure 1a).

First, homemade CF_MP_ (Hunan University, Changsha, China) was used as a reinforcement and heat carrier of the composites in both X and Y directions by yarn winding, and commercially available CF_PAN_ (T700, Toray, Tokyo, Japan) was used as the reinforcement in the Z direction by puncture in order to improve the mechanical properties of the composite (Figure 1). Table 1 shows the characteristics of the carbon fibers in the C_MP_/C and C_PAN_/C composites. The density of the preform is roughly 0.9 g·cm^−3^.

Second, 3D CF_MP_ preform filling or densification was accomplished by a combination of chemical vapor infiltration (CVI) and polymer infiltration and pyrolysis (PIP). CVI densification was performed in a hot-wall furnace, and the pressure was 3.0 kPa. The reaction was at 950 °C using C_3_H_6_ as the carbon precursor gas and nitrogen as the carrier gas at a volumetric ratio of 2:5. The density of the sample was about 1.6 g·cm^−3^ after CVI for 80 h. Subsequently, the impregnation and solidification processes were repeated 4–6 times using furan resin as the precursor. Then, carbonization was carried out in the furnace at 900 °C in a nitrogen atmosphere. After PIP and CVI, pyrolytic carbon (PyC) was formed into the preform and wrapped around the carbon fibers. The 3D C_PAN_/C composite using CF_PAN_ as reinforcement in all directions was prepared by the same method and was used as the control.

Finally, after graphitization at 3000 °C under an argon atmosphere, the 3D high-thermal-conductive C_MP_/C composite and low-thermal-conductive C_PAN_/C composite with a density of 1.82–1.84 g/cm^3^ were obtained.

The SiC coating was fabricated on the surface of the samples by the same chemical vapor reaction (CVR) to develop ablation resistance. Then, the C_MP_/C and C_PAN_/C composites were ground with SiC papers (800 grit), ultrasonically cleaned and dried, and the SiC coating was fabricated on the composites by the CVR process. The SiO_2_/Si mixture was heated at 1500–1800 °C to produce SiO gas. The CVR SiC coating was prepared by a reaction between the SiO gas and the C_MP_/C or C_PAN_/C composite at 2200 °C in a graphite furnace, to obtain SiC-coated samples.

### 2.2. Ablation and Oxidation Tests

The ablation resistance of the C_MP_/C and C_PAN_/C composite samples (Ø5 × 35 mm) was tested by water plasma equipment (Multiplaz 3500, muzzle inner diameter 3–5 mm). The ablation direction of the flame was parallel to the X (Y) direction of the preform (Figure 1). The distance between the muzzle and the composite was 10 mm, and the composite was ablated for 120 s. The maximum temperature of the plasma was about 2300 °C, as measured by an optical pyrometer. The surface temperature (hot side) of the specimen was monitored by a noncontact infrared pyrometer, while the back temperature (cool side) was measured by the thermocouple. The average ablation rates were calculated using five samples. The mass ablation rate was calculated using Equation (1):

R_m_ = ∆m·S^−1^·t^−1^ (1)

where R_m_ is the mass ablation rate (mg·cm^−2^·s^−1^), ∆m is the mass change of the sample (mg), S is the surface area of the coating (cm^2^), and t is the ablation time (s).

The linear ablation rate was calculated by Equation (2):

R_l_ = ∆l·t^−1^ (2)

where R_l_ is the linear ablation rate (µm·s^−1^), ∆l is the length change of the sample (µm), and t is the ablation time (s).

The static oxidation behavior of the samples was tested in a muffle furnace. After being heated to 1500 °C for 10 min in the furnace, the samples cooled down to room temperature. Air was used as the oxidizing gas in the furnace, and the cooling rate was 100 °C·min^−1^.

### 2.3. Material Characterization

The structure and morphology of the samples were analyzed by scanning electron microscopy (SEM; FEI Nova Nano SEM230, Hillsboro, OR, USA), and the phase composition of the samples was characterized by X-ray diffraction (XRD; Rigaku Dmax 2550VB + 18 KW, Tokyo, Japan).

The thermal conductivity of the carbon fibers was obtained by an indirect test method. After measuring the axial electrical resistivity of the carbon fibers with the four-probe method, the thermal conductivity values were calculated using Equation (3):

λ = 1261/ρ(3)

where λ is the thermal conductivity and ρ is the specific resistance.

The thermal diffusivity of the composites was tested by a laser flash diffusivity apparatus (NETZSCH, Selb, Germany). The thermal conductivity was calculated using Equation (4):

k = α·c_p_·ρ (4)

where α is the thermal diffusion coefficient, c_p_ is the specific heat at constant pressure, and ρ is density.

## 3. Results and Discussion

### 3.1. Microstructure of the Composites

**Figure 2:**
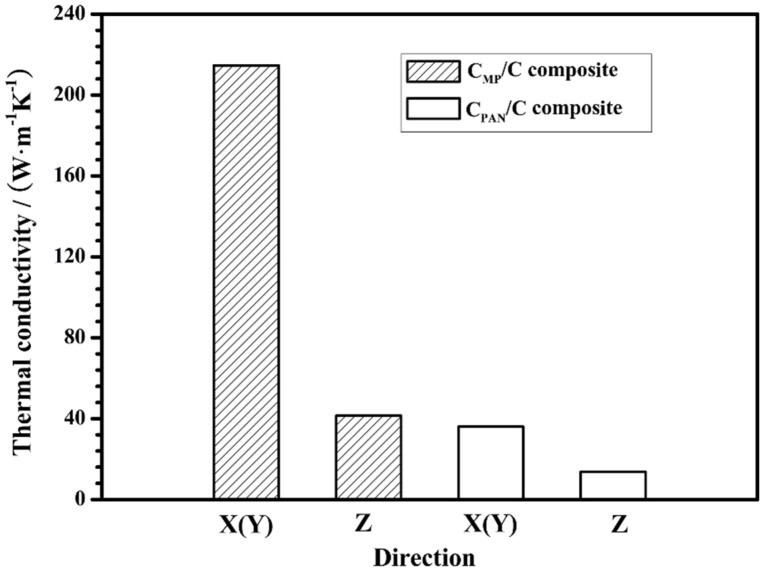
Thermal conductivity of C_MP_/C and C_PAN_/C composites. C_PAN,_ polyacrylonitrile (PAN)-based carbon fiber.

The room-temperature thermal diffusion of the C_MP_/C composite is closely related to the composition, structure, and arrangement orientation of the carbon fibers and carbon matrix. The room-temperature thermal conductivity of the homemade C_MP_/C and C_PAN_/C composites is shown in Figure 2. It is known that the orientation and size of the graphite layer play important roles in the thermal conductivity of carbon fibers. The homemade CF_MP_ had a larger graphite crystallite size, higher preferred orientation degree along the axis, and fewer crystallite defects, so it had higher thermal conductivity than CF_PAN_. The thermal conductivity of CF_MP_ and the CF_PAN_ is about 700 W·m^−1^·K^−1^ and 10 W·m^−1^·K^−1^, respectively (Table 1). Figure 2 shows that the thermal conductivity of the C_MP_/C composite (218.2 W·m^−1^·K^−1^) is evidently higher in the X and Y directions than that of the C_PAN_/C composite (36.1 W·m^−1^·K^−1^). It is speculated that the C_MP_/C composite can transfer heat from the hot side to the cool side more efficiently than the C_PAN_/C during ablation in the X or Y direction. In addition, because CF_MP_ in the X (Y) direction has higher thermal conductivity and a higher volume fraction than CF_PAN_ in the Z direction (Table 1), the thermal conductivity of the C_MP_/C composite in the X (Y) direction is higher than that in the Z direction.

**Figure 3:**
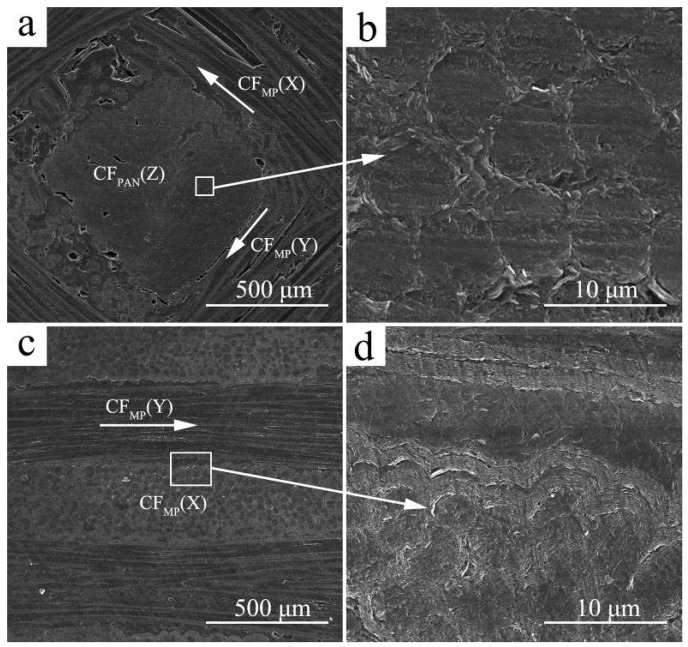
Microstructure of C_MP_/C composite: (**a**) X–Y plane; (**b**) CF_PAN_ in the Z direction; (**c**) Y–Z plane; (**d**) high-magnification image.

Figure 3 shows the microstructure of the C_MP_/C composite. It can be seen that the C_MP_/C composite contains a small amount of cracks, voids, and debonded fiber/matrix interfaces. However, the composite is almost compact after densification (Figure 3a). Figure 3b shows that the round-shaped CF_PAN_ in the Z direction is coated with PyC. The thickness of the PyC layer in CF_PAN_ is less than that of the CF_MP_ due to smaller gaps in CF_PAN_. The arrows in Figure 3a,c indicate the direction of the CF_MP_ bundle in the composite. Compared to CF_PAN_, with thermal conductivity of about 10 W·m^−1^·K^−1^, it is evident that CF_MP_ has higher thermal conductivity, reaching to above 700 W·m^−1^·K^−1^, which can contribute to the highly oriented graphitic structure along the fiber axis, larger crystal size, and more perfect crystallinity of CF_MP_ [13]. Thus, the C_MP_/C composite has higher thermal conductivity in the X (Y) direction. High-magnification images indicate good interface bonding between PyC and CF_MP_, and resin carbon can also be found in the gaps among the PyC layers.

**Figure 4:**
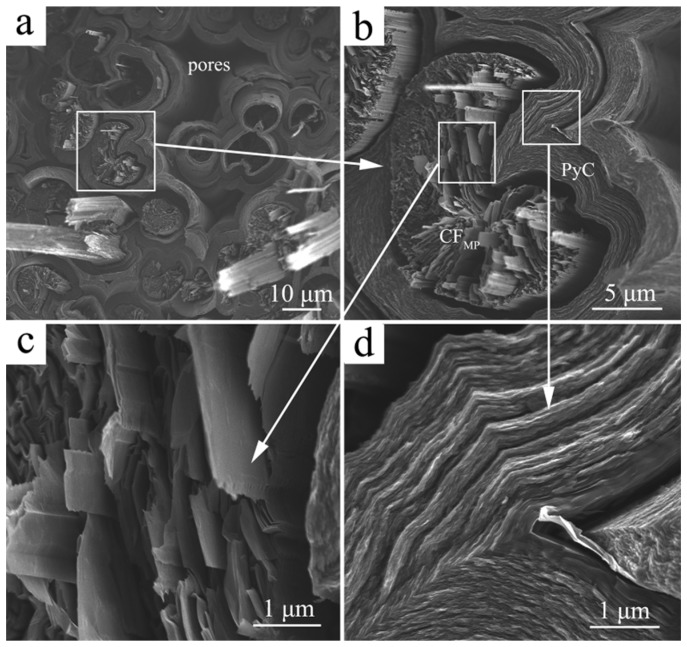
SEM images of fracture section of C_MP_/C composite: (**a**) low-magnification image; (**b**–**d**) high-magnification images of PyC-coated CF_MP_, highly preferentially oriented crystal structure in CF_MP_ (**c**) and PyC (**d**). The white arrows show the regions at high magnification.

To further understand the influence of the microstructure of C_MP_/C composite on its ablation behavior, its fracture morphology was investigated (as shown in Figure 4). The location of this particular fracture in Figure 4 refers to the region of the dashed white lines in Figure 3c. Carbon fiber pull-out and interface debonding can be observed after rupture failure of the C_MP_/C composite (Figure 4a). It is interesting to find that the open wedge crack texture of the round-shaped CF_MP_ is observed in the C_MP_/C composite (Figure 4b). It is reported that the linear domain units in CF_MP_ induced circumferential shrinkage at the spinning and further heat-treatment steps, leading to the formation of the open crack [13,25]. Figure 4c indicates that CF_MP_ is composed of a highly graphitic flat-layered structure radially oriented in the transverse section. The thermal conductivity of the carbon materials can be calculated according to the following formula:

λ = 1/3CνL(5)

where λ is the thermal conductivity, ν is the propagation velocity of the phonon, and L is the free path of the phonon. CF_MP_ has well-developed graphene sheets and a highly preferentially oriented crystal structure along the longitudinal and radial directions, which can provide fast thermal diffusion in phonon heat conduction. After graphitization, the layered structure of PyC is also formed (Figure 4d). Because the diameter of CF_MP_ is larger and the gas precursor has a larger diffusion space in the preform during the CVI process, the PyC layer around CF_MP_ is thicker than that around CF_PAN_.

### 3.2. Ablation Behavior of C_MP_/C Composite

**Figure 5:**
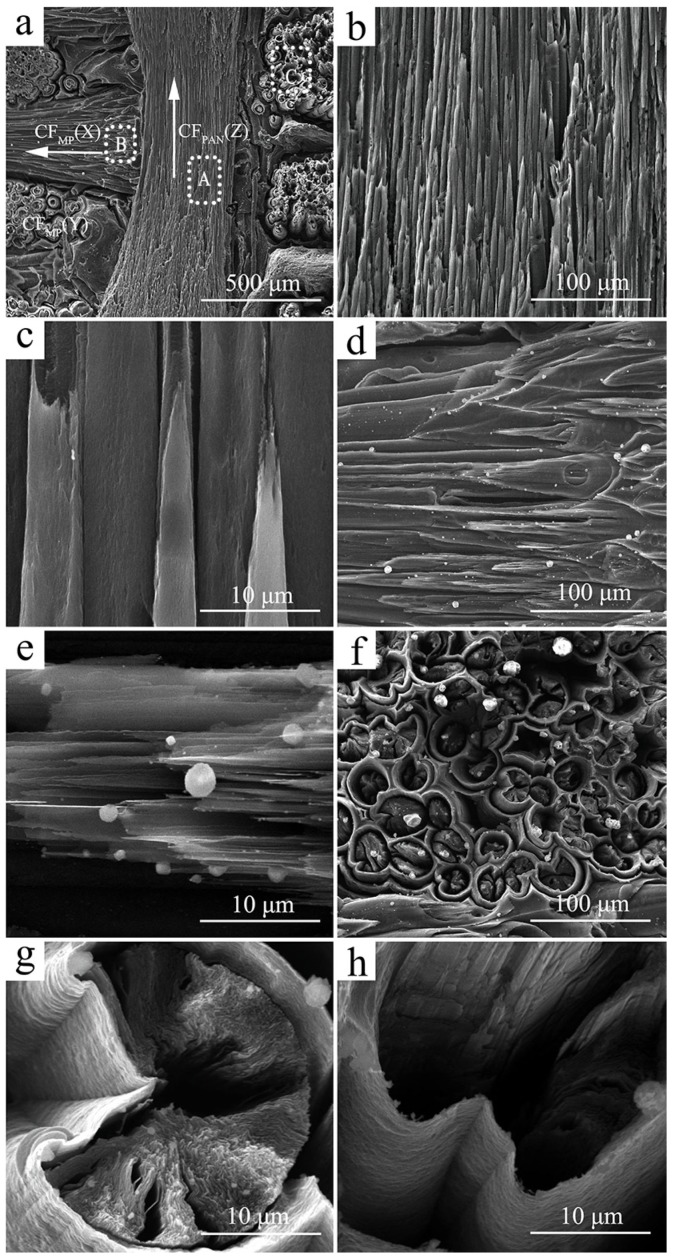
SEM images of C_MP_/C composite after ablation: (**a**) microstructure in the ablation center; (**b**) low-magnification image of CF_PAN_ in the Z direction (region A in (**a**)); (**c**) high-magnification image of CF_PAN_ in the Z direction (region A in (**a**)); (**d**) low-magnification image of CF_MP_ in the Y direction (region B in (**a**)); (**e**) high-magnification image of CF_MP_ in the Y direction (region B in (**a**)); (**f**) low-magnification image of CF_MP_ in the X direction (region C in (**a**)); (**g**) high-magnification image of CF_MP_ in the X direction (region C in (**a**)); (**h**) interface between PyC layers after ablation (region C in (**a**)). The white arrows in (**a**) show the directions of the CF_PAN_ and CF_MP_.

Figure 5 shows SEM images of the C_MP_/C composite (X–Z plane) after ablation. The ablated surface exhibits a rough and porous morphology and is mainly composed of ablated carbon fiber and matrix. It can be seen that both CF_MP_ in the X (Y) direction and CF_PAN_ in the Z direction are ablated (Figure 5a), but they show different ablation characteristics. CF_PAN_ becomes needle-shaped after ablation (Figure 5b), which has been reported by many studies [24,25,26]. Because CF_PAN_ has a turbostratic structure with physical entanglements and covalent cross-links, its cross-section after ablation looks like a homogeneous structure (Figure 5c) [15,16]. No peeling of the graphene sheet is observed. Compared to CF_PAN_, CF_MP_ shows a wedge shape after ablation (Figure 5d). The layered structure in the fiber axis direction is formed after ablation, as shown in Figure 5e, suggesting that the mass loss preferentially takes place at the edges of layers or among layers because of the radial texture of CF_MP_. Figure 5f exhibits the ablated morphology of CF_MP_ in the X direction, showing that the matrix (PyC) around the fiber bundles is eroded and forms a shell shape, and then is stripped off by the plasma flame. Before ablation, the boundary inside the carbon matrices and the CF/PyC interface presents good compatibility, and there is no PyC matrix microcracking or obvious interfacial debonding (Figure 3d). However, after ablation, many gaps or cracks are formed in the PyC matrix, the CF bundle, and the interface between them (Figure 5h). These indicate that ablation and oxidation begin at the CF/PyC interface and the boundary inside the PyC matrix, which are much easier to oxidize than the PyC matrix because of the more active sites in carbon nets. It is noted that many ablated defects, such as gaps or cracks, are also formed in the radial direction of CF_MP_ (Figure 5g).

**Figure 6:**
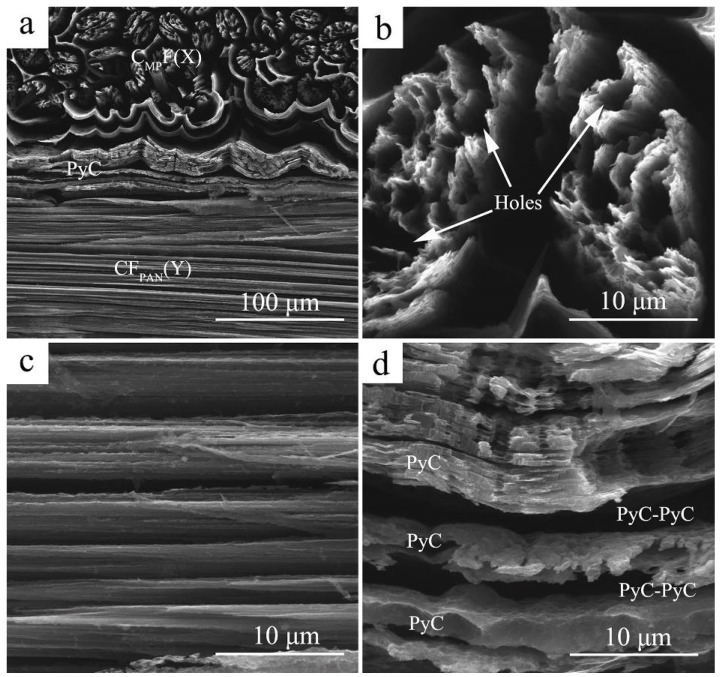
Microstructure of C_MP_/C composite after oxidation: (**a**) microstructure of the Y–Z plane; (**b**) CF_MP_ in the X direction; (**c**) CF_MP_ in the Y direction; (**d**) interface between PyC layers.

In order to better analyze the ablation behavior of C_MP_/C composite, we performed an oxidation test (Figure 6). It is observed that oxidation preferentially occurs at the CF/PyC interface, which is similar to ablation. When oxygen diffuses from the outer to the inner part of the C_MP_/C composite, the CF/PyC interface and boundary inside the matrix oxidize much more easily than the PyC matrix because there are more active sites in carbon nets (Figure 6a). The cross-section of CF_MP_ after the oxidation test shows a more homogeneous morphology compared to the ablation test (shown in Figure 5g). Many small holes formed by oxidation can be observed in Figure 6b. The highly conductive CF_MP_ in this study has a radial structure and weaker bonding among the highly oriented graphene layers. It can be speculated that the carbon atoms at these active sites may preferentially react with oxygen, leading to the radial carbon fragments formed by oxidation being easily stripped away from the CF_MP_ (Figure 5g). Figure 6c shows the oxidation characteristics in the length direction of CF_MP_. Many slits are observed after oxidation, which can provide oxygen diffusion paths in the CF_MP_ during oxidation. Figure 6d shows the oxidation characteristics in the carbon matrix. It can be seen that the PyC/PyC interface is more easily oxidized than the PyC itself. Slits are formed in the PyC/PyC interface, and some small holes are observed on the surface of the PyC matrix, indicating that the PyC/PyC interface presents higher chemical reactivity than the PyC matrix itself during oxidation. In addition, the oxidation and ablation tests showed that a thermal protective coating is necessary to prevent the C_MP_/C composite from being damaged in the oxygen atmosphere. 

**Figure 7:**
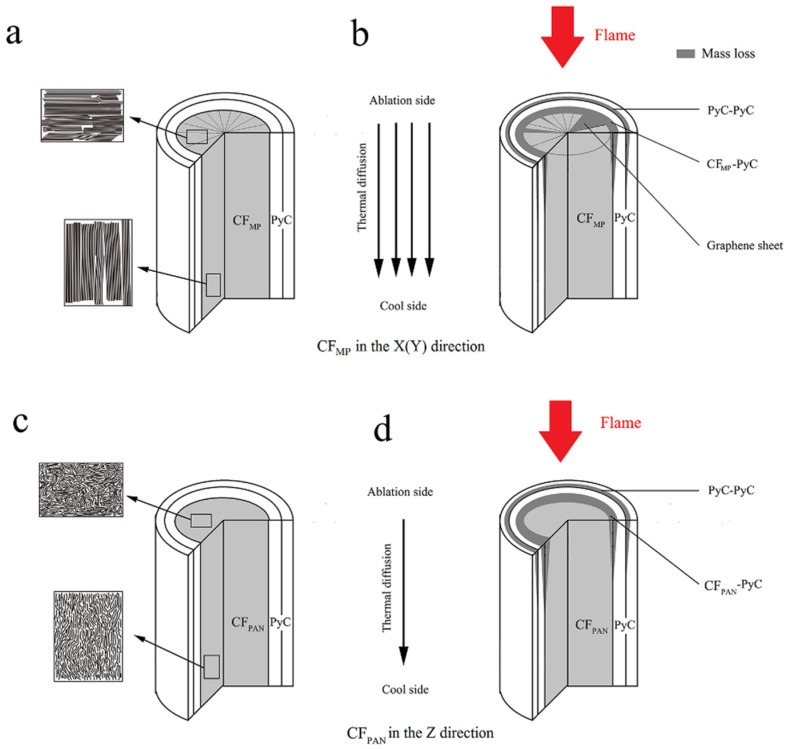
Ablation behavior of C_MP_/C composite in different directions: (**a**) CF_MP_ in the X (Y) direction before ablation; (**b**) CF_MP_ in the X(Y) direction after ablation; (**c**) CF_PAN_ in the Z direction before ablation; (**d**) CF_PAN_ in the Z direction after ablation.

The ablation behavior of the C_MP_/C composite is mainly influenced by the oxidation reaction and the mechanical scouring caused by the plasma flame. The ablation behavior can be explained as follows (Figure 7): On the one hand, the oxidation reaction of the C_MP_/C composite refers to the heterogeneous reaction between the oxygen and the carbon phase, including carbon fiber and carbon matrix. The CF/PyC and PyC/PyC interfaces are preferentially oxidized and damaged due to the cracks and debonding defects. The PyC matrices around the CF bundle are burned into a shell shape, while the PyC matrices among the ablated CF are burned off. On the other hand, CF_MP_ and CF_PAN_ exhibit different ablation behaviors due to their different structural characteristics. CF_PAN_, with physical entanglements and covalent cross-links of turbostratic structure, becomes needle-shaped after ablation (Figure 5b). Compared to the homogeneous ablated structure of CF_PAN_, CF_MP_—with a highly preferentially oriented crystal structure—shows a wedge shape after ablation. The carbon phases in or between the edges of the layers of the highly graphitic flat-layered structure are more easily oxidized and stripped away from the CF_MP_ during ablation. Therefore, the mass loss is observed in the radial direction.

### 3.3. Ablation Behavior of SiC-Coated C_MP_/C Composite

**Figure 8:**
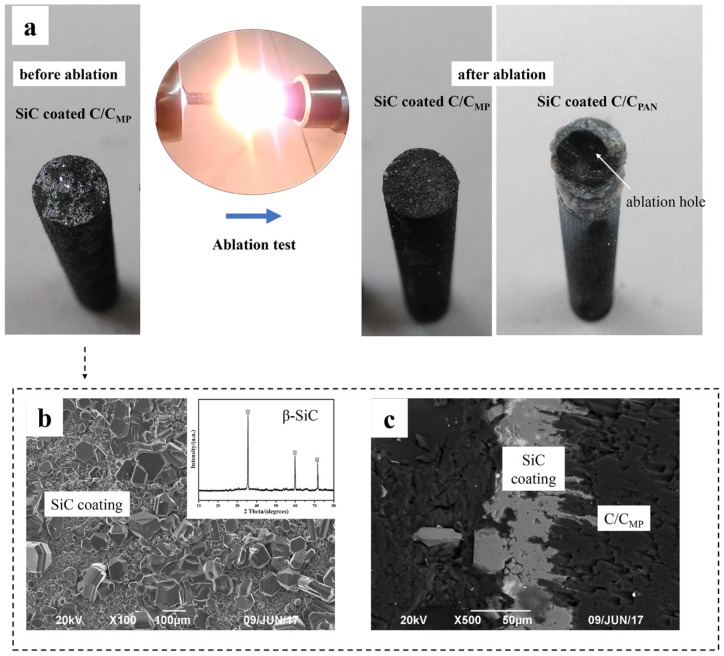
Microstructure of SiC coating: (**a**) photos of SiC-coated samples before and after ablation tests; (**b**) SEM image of the surface of the coating; (**c**) SEM image of the cross-section of the coating.

In order to further optimize the ablation resistance of the C_MP_/C composite, SiC coating was fabricated on the surface of samples by CVR (Figure 8). Figure 8b,c shows SEM images of the surface and cross-section of the coatings after CVR. The SiC coating surface seems to be coarse and rubble-like before ablation. The XRD pattern (Figure 8b) indicates that the coating is composed of β-SiC, which has three obvious characteristic peaks at 35.7°, 60.1°, and 71.8° ((111), (220), and (311), respectively, in a Face-Centered Cubic lattice). Figure 8c shows that the thickness of the SiC coating is about 50–80 μm, and there is no obvious crystal boundary in the cross-section (Figure 8c).

**Figure 9:**
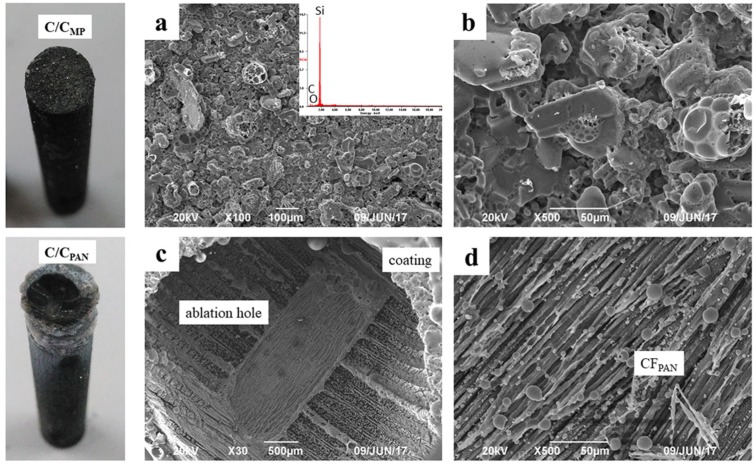
Microstructure of samples after ablation for 120 s: (**a**) low-magnification SEM image of ablation center on surface of SiC-coated C_MP_/C composite; (**b**) high-magnification image of (**a**); (**c**) low-magnification SEM image of the ablation center on the surface of SiC-coated C_PAN_/C composite; (**d**) high-magnification image of (**c**).

Photographs of the composites after 120 s of ablation are shown in Figure 9. The coating in the ablation center of C_MP_/C composite was not destroyed after ablation. The SiC grains were covered with a glass layer, which was identified as being SiO_2_ by EDS results (Figure 9a). It is worth noting that the ablation temperature (2300 °C) exceeded the boiling point of SiO_2_, but the SiC was not evidently corroded during ablation; only a few pitting corrosion features were observed on the coating surface. In comparison, coating in the ablation center of C_MP_/C composite was stripped off and the carbon fibers were ablated. Although some ceramic oxide residues were observed on the surface, they could not prevent the carbon phase from erosion. Table 2 shows the ablation rates of the C_PAN_/C and C_MP_/C composites with and without SiC coating. It can be concluded that without SiC coating, both composites show similar ablation rates during the test because the carbon phases are more easily oxidized and then stripped from the composites. Thermal conductivity does not have an obvious effect on the thermal protection. However, the SiC-coated C_MP_/C composite shows better ablation resistance than the SiC-coated C_PAN_/C composite after a 120 s ablation test. The mass and linear ablation rates of the SiC-coated C_PAN_/C composite are 13.57 μm·s^−1^ and 1.44 mg·(cm^−2^·s^−1^), respectively, and those of the SiC-coated C_MP_/C composite are only 0.52 μm·s^−1^ and 0.19 mg·(cm^−2^·s^−1^), respectively. Thermal conductivity is believed to play a significant role in decreasing the hot-side temperature and protecting the SiC coating from corrosion by the flame.

**Figure 10:**
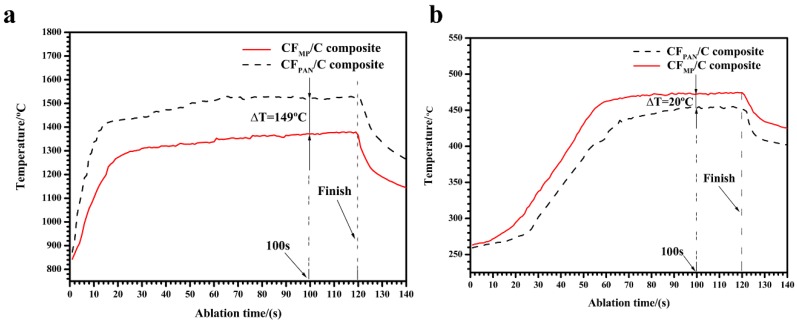
Temperature curves of samples at (**a**) ablation center and (**b**) cool side under the same ablation conditions.

Figure 10a shows the surface temperature curves of the C_MP_/C and C_PAN_/C composites. It shows that the thermal conductivity of the composite has an influence on its surface temperature, because the composite with higher thermal conductivity allows more heat to be conducted from heating zones (sample surface) to colder zones (sample back), and then be dissipated. It can be seen from Figure 10a that the surface temperature of the C_MP_/C composite is about 1370 °C at 100 s, which is lower than the flame temperature (over 2300 °C) due to its high emissivity and thermal conductivity. The surface temperature of the C_PAN_/C composite increases rapidly to about 1427 °C at 20 s, whereas that of the C_MP_/C composite increases more slowly. After 100 s of ablation, the surface temperature of the C_MP_/C composite is 149 °C lower than that of the C_PAN_/C composite, indicating that CF_MP_ can effectively dissipate heat away from the hot side (ablation center) to the relatively cool side (sample back). It is believed that CF_MP_ is composed of a highly preferentially oriented crystal structure in the longitudinal direction, which can provide fast thermal diffusion in phonon heat conduction. The straight and continuous CF_MP_ can effectively decrease the surface temperature by rapidly transferring the heat to the back side, and then radiate the heat around the environment. Because the thermal conductivity of the C_MP_/C composite (218.2 W·m^−1^·K^−1^) is much higher than that of the C_PAN_/C composite (36.1 W·m^−1^·K^−1^) along the ablation direction (Figure 1 and Figure 2), it can be concluded that more heat in the ablation center of the C_MP_/C composite is dissipated away under the same conditions. Figure 10b shows that the temperature of the C_MP_/C composite on the cool side (sample back) increases more than that of the C_PAN_/C composite. At 100 s, the cool-side temperature of the C_MP_/C composite is 20 °C higher than that of the C_PAN_/C composite. The result also indicates that more heat can be conducted from the ablation center (hot side) to the sample back (cool side) when C_MP_ with high conductivity is used in the composite. Based on this analysis, it can be concluded that the high conductivity of the C_MP_/C composite (CF_MP_) plays a positive role in its ablation resistance.

## 4. Conclusions

The prepared C_MP_/C composite has higher thermal conductivity in the X and Y directions. After the ablation test, CF_PAN_ becomes needle-shaped, while CF_MP_ shows a wedge shape. The fiber/matrix and matrix/matrix interfaces are preferentially oxidized and damaged during ablation. If there is no SiC coating, the C_MP_/C and C_PAN_/C composites show similar ablation rates during ablation. After being coated by SiC coating, thermal conductivity is believed to play a significant role in decreasing the hot-side temperature and protecting the coating from corrosion by the flame. The SiC-coated C_MP_/C composite shows better ablation resistance than the SiC-coated C_PAN_/C composite after a 120 s ablation test. The mass and linear ablation rates of the SiC-coated C_MP_/C composite are only 0.52 μm·s^−1^ and 0.19 mg·cm^−2^·s^−1^, respectively. The result indicates that the C_MP_/C composite can effectively decrease the hot side temperature by rapidly conducting heat to the cool side, which can improve its ablation resistance.

This work is focused on the microstructural features and ablation properties of C_MP_/C composite. Actually, the finite element model is believed to be an effective method to analyze its thermal conduction mechanism and ablation behavior. Further research on this advanced computational method is ongoing in this program.

## Figures and Tables

**Table 1:** Characteristics of carbon fiber in C_MP_/C composite. CF_MP_, mesophase pitch-based carbon fiber; CF_PAN_, polyacrylonitrile carbon fiber.

	Thermal Conductivity (W·m^−1^·K^−1^)	Volume Fraction (%)	Direction
**CF_MP_**	≥700	36	X, Y
**CF_PAN_**	~10	8–10	Z

**Table 2:** Ablation rates of samples after ablation under air atmosphere for 120 s.

Sample	Linear Ablation Rate (μm·s^−1^)	Mass Ablation Rate (mg·cm^−2^·s^−1^)
SiC-coated C_MP_/C	0.52	0.19
SiC-coated C_PAN_/C	13.57	1.44
C_MP_/C	28.21	12.24
C_PAN_/C	26.45	11.31

## Abbreviations

C/C composite	The carbon-fiber-reinforced carbon matrix (C/C) composite
CF	carbon fiber
PAN	polyacrylonitrile
CF_PAN_	PAN-based carbon fiber
CF_MP_	mesophase pitch-based carbon fiber
C_MP_/C composite	C/C composite prepared using the CF_MP_ in the X (Y) direction and the CF_PAN_ in the Z direction in this study
C_PAN_/C composite	C/C composite prepared using the CF_PAN_ in all directions
CVI	chemical vapor infiltration
PIP	polymer infiltration and pyrolysis
CVR	chemical vapor reaction
